# Assessing advances in three decades of clinical antiretroviral therapy on the HIV-1 reservoir

**DOI:** 10.1172/JCI183952

**Published:** 2024-11-29

**Authors:** Irene González-Navarro, Víctor Urrea, Cristina Gálvez, Maria del Carmen Garcia-Guerrero, Sara Morón-López, Maria C. Puertas, Eulàlia Grau, Beatriz Mothe, Lucía Bailón, Cristina Miranda, Felipe García, Lorna Leal, Linos Vandekerckhove, Vincent C. Marconi, Rafick P. Sekaly, Bonaventura Clotet, Javier Martinez-Picado, Maria Salgado, the NIH Reversing Immune Dysfunction for HIV-1 Eradication (RID-HIV) Collaboratory Group

**Affiliations:** 1 IrsiCaixa, Badalona, Barcelona, Spain.; 2 Department of Cellular Biology, Physiology and Immunology, Faculty of Medicine, Autonomous University of Barcelona, Barcelona, Spain.; 3 Biomedical Research Networking Center on Infectious Diseases (CIBERINFEC), Carlos III Health Institute (ISCIII), Madrid, Spain.; 4 Germans Trias i Pujol Research Institute (IGTP), Campus Can Ruti, Badalona, Spain.; 5 Department of Infectious Diseases and Fundació Lluita contra les Infeccions, Germans Trias i Pujol University Hospital (HUGTiP), Badalona, Spain.; 6 Chair in Infectious Diseases and Immunity, University of Vic – Central University of Catalonia (UVic-UCC), Vic, Spain.; 7 Department of Medicine, Autonomous University of Barcelona, Barcelona, Spain.; 8 Infectious Diseases Department Hospital Clinic, University of Barcelona, Barcelona, Spain.; 9 HIV Cure Research Center, Department of Internal Medicine and Pediatrics, Ghent University Hospital, Ghent University, Ghent, Belgium.; 10 Division of Infectious Diseases, Emory University School of Medicine, Atlanta, Georgia, USA.; 11 Department of Global Health, Rollins School of Public Health, Emory University, Atlanta, Georgia, USA.; 12 Emory Vaccine Center, Emory University, Atlanta, Georgia, USA.; 13 The Atlanta Veterans Affairs Medical Center, Decatur, Georgia, USA.; 14 Pathology Advanced Translational Research Unit (PATRU), Department of Pathology and Laboratory Medicine and; 15 Winship Cancer Institute, Emory University School of Medicine, Atlanta, Georgia, USA.; 16 Catalan Institution for Research and Advanced Studies (ICREA), Barcelona, Spain.; 17 The NIH Reversing Immune Dysfunction for HIV-1 Eradication (RID-HIV) Collaboratory group is detailed in the Supplemental Acknowledgments.

**Keywords:** AIDS/HIV, Virology, Drug therapy, Medical statistics, Molecular pathology

## Abstract

**BACKGROUND:**

Antiretroviral therapy (ART) has improved the clinical management of HIV-1 infection. However, little is known about how the latest ART recommendations affect the heterogeneity of the HIV-1 reservoir size.

**METHODS:**

We used a complete statistical approach to outline parameters underlying the diversity in HIV-1 reservoir size in a cohort of 892 people with HIV-1 (PWH) on suppressive ART for more than 3 years. Total HIV-1–DNA levels were measured in PBMCs using digital droplet PCR (ddPCR).

**RESULTS:**

We classified 179 (20%) participants as being low viral reservoir treated (LoViReT) (<50 HIV-1–DNA copies/10^6^ PBMCs). Twenty variables were collected to explore their association with the LoViReT phenotype using machine learning approaches. LoViReT status was closely associated with higher nadir CD4, lower zenith pre-ART viral load, lower CD4 recovery, shorter time from diagnosis to undetectable viral load, and initiation of treatment with an integrase inhibitor–containing (InSTI-containing) regimen. Initiation of ART with any InSTI was also linked with a shorter time to undetectable viremia. Locally estimated scatterplot smoothing (LOESS) regression revealed a progressive reduction in the size of the HIV-1 reservoir in individuals who started ART after 2007. Similarly, a higher nadir CD4 and a shorter time to undetectable viremia were observed when treatment was initiated after that year.

**CONCLUSION:**

Our findings demonstrate that the progressive implementation of earlier, universal treatment at diagnosis and the use of InSTIs affected the size of the HIV-1 reservoir. Our work shows that effective management of infection is the first step toward reducing the reservoir and brings us closer to achieving a cure.

**FUNDING:**

NIH; Division of AIDS at the National Institute of Allergy and Infectious Diseases (NIAID), NIH; Merck Sharp & Dohme.

## Abstract 2

In this manuscript, we show that the progressive implementation of earlier, universal treatment at diagnosis and the use of InSTIs affect the size of the HIV-1 reservoir in the population of PWH.

## Graphical abstract

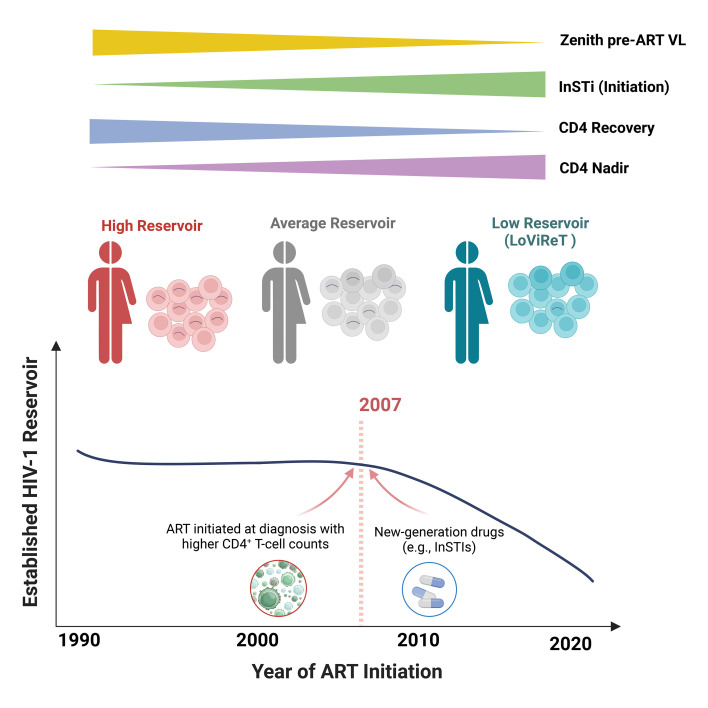

## Introduction

Antiretroviral therapy (ART) constitutes a major advance in the clinical management of the HIV-1 pandemic. Modifying the natural course of the infection has substantially improved the life expectancy of people with HIV-1 (PWH) (1). However, despite achieving undetectable plasma viral loads (VLs), effective ART does not completely eliminate the virus, which persists as a residual latent infection in resting CD4^+^ T cells. This HIV-1 reservoir contributes to ongoing inflammation and immune activation, favoring a rapid relapse of viremia if ART is interrupted (2).

The substantial improvement in medical surveillance and treatment of HIV-1 infection during the past 2 decades has generated novel and safer antiretroviral agents that increase adherence and improve disease prevention and control (3). Hence, first-line regimens incorporating newly approved potent protease inhibitors (PIs) and integrase strand transfer inhibitors (InSTIs) have been increasingly recommended. Updated clinical guidelines have also implemented a “treatment-for-all” approach, advocating for the initiation of ART regardless of disease stage or CD4^+^ T cell count, thereby promoting earlier initiation of treatment (4). Additionally, community-based centers have played a pivotal role in establishing test-and-treat strategies during acute and early phases of HIV-1 infection, facilitating rapid detection of HIV-1, and expediting linkage to ART programs or immediate initiation of ART, thus enhancing the quality of life for PWH (5). However, the effect of these advances on reservoir size in PWH receiving ART requires further exploration.

One of the most widely accepted hypotheses is that earlier initiation of ART is a crucial factor in minimizing the size of the viral reservoir (6). We previously reported that individuals who commenced ART during chronic infection can also harbor a relatively small reservoir (7). In that study, we described a new phenotype of PWH on ART (treated during acute or chronic infection) who exhibit low levels of HIV-1–DNA in both the periphery and anatomical sanctuaries and that are referred to as low viral reservoir treated (LoViReT) (7, 8). We observed that these individuals had lower levels of HIV-1–DNA prior to initiation of ART, followed by a more pronounced decay after 1.5 and 5 years of treatment, indicating that the LoViReT phenotype is affected by host and clinical factors (7). In our previous work, we analyzed viral reservoirs from individuals who initiated ART between the late 1990s and 2011, thus obviating the potential effects of the newer drugs (e.g., InSTIs) and recommendations (e.g., treatment regardless of CD4^+^ T cell counts) on the analysis (7).

In the current study, we expanded our screened cohort until it reached 892 treated PWH using total HIV-1–DNA quantification in PBMCs to characterize the established reservoir of participants initiating treatment up to 2019. We used an exhaustive statistical approach to assess clinical management of HIV-1 infection over a 30-year period. We were able to perform a more comprehensive analysis of features that may be associated with the reduction in the ART-established viral reservoir (e.g., treatment regimens, cell counts, VL, HIV-1 tropism, time from diagnosis to initiation of ART).

## Results

### Characteristics of the study population.

**Figure 1:**
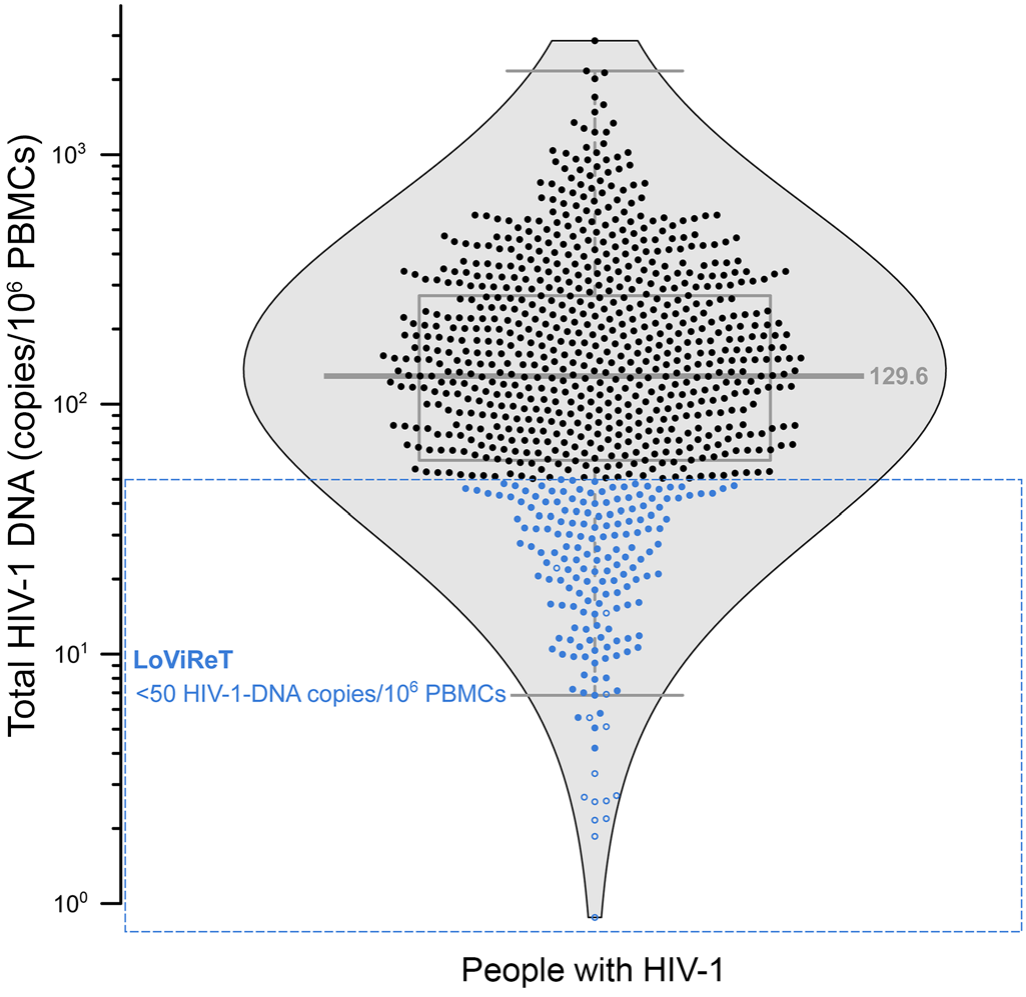
Distribution of the HIV-1 reservoir. Distribution of total HIV-1–DNA for the 892 volunteers screened by ddPCR. LoViReT participants (<50 HIV-1–DNA copies/10^6^ PBMCs) are shown in blue. Open symbols indicate values that fall below the detection limit; in these scenarios, the limit of detection varied according to sample/volume input.

We analyzed 892 PWH who had been receiving suppressive ART for a median of 7.5 years [IQR, 4.9–15.2]. Proviral levels followed a normal log_10_ distribution, with a median of 129.6 HIV-1–DNA copies/10^6^ PBMCs [IQR, 59.6–272.1] for the entire population (Figure 1). A total of 179 (20%) participants were identified as LoViReTs (defined as <50 HIV-1–DNA copies/10^6^ PBMCs), including 14 (1.6%) who harbored proviral levels below the limit of detection for the 2 primer/probe sets used (Figure 1).

The clinical characteristics of the study population and the LoViReT subgroup are summarized in Table 1. We observed statistically significant differences between the LoViReT group and non-LoViReT group for many of the variables included in the analyses. However, we noticed that variables such as time from HIV-1 diagnosis to sampling (timeDtoS) and time from initiation of ART to sampling (timeARTtoS), as well as the CD4/CD8 ratio and CD4^+^ T cell counts were correlated with each other (Supplemental Figure 1; supplemental material available online with this article; https://doi.org/10.1172/JCI183952DS1). Consequently, assessment of the relevance of each factor in identifying LoViReT status proved challenging. To reduce confounding factors, we performed a multiparametric analysis, in which we characterized the variables that are strongly associated with the LoViReT phenotype.

### The LoViReT phenotype is strongly associated with higher nadir CD4 counts, lower levels of pre-ART viremia, and shorter time to viral suppression.

We evaluated over 20 demographic, virologic, and immunologic clinical parameters (Table 1 and Supplemental Figure 2) and analyzed the initiation and duration of treatment with 30 antiretroviral drugs, either individually or grouped into 6 distinct families, using random forest machine learning algorithms to identify factors associated with low reservoirs that contribute to the LoViReT phenotype.

**Figure 2:**
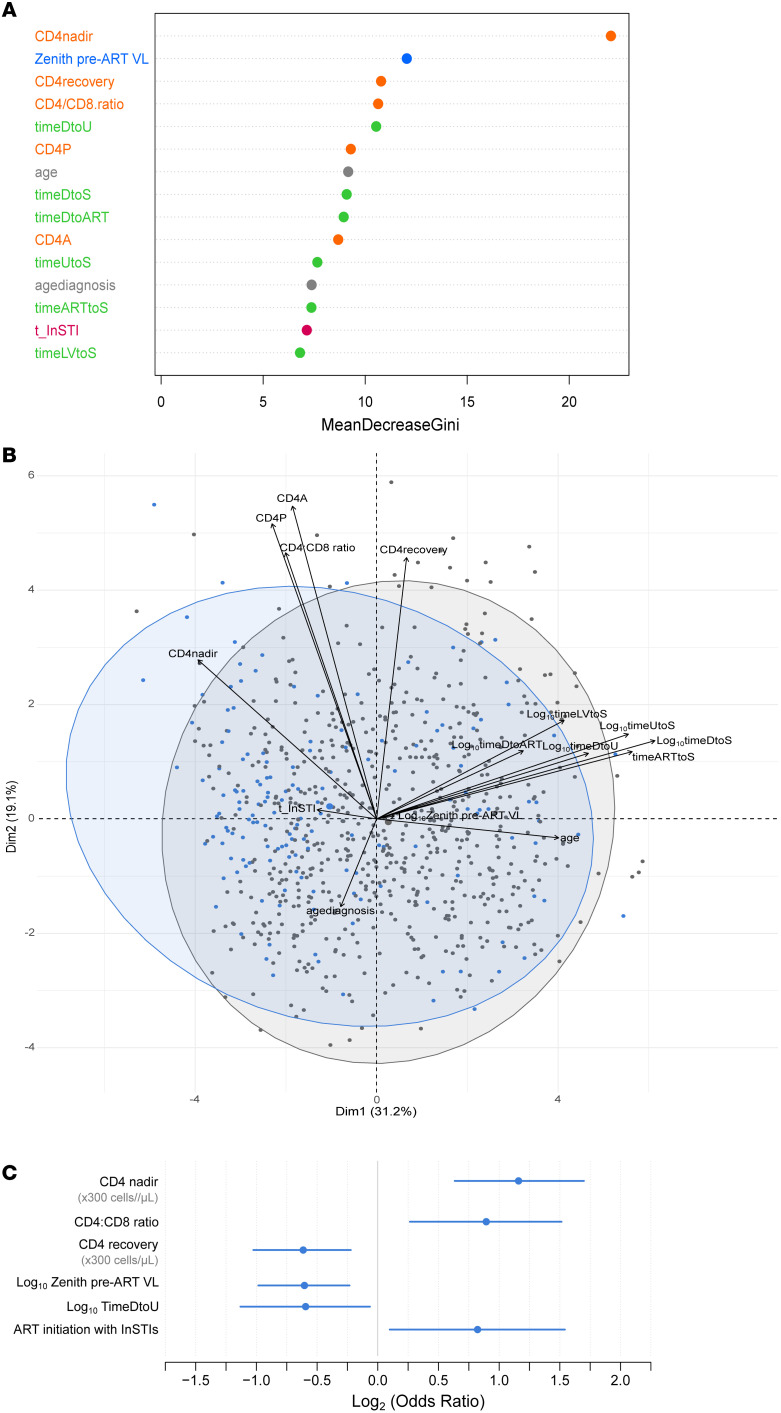
Multivariate analysis to characterize the LoViReT phenotype. (**A**) Representation of the top 15 low viral reservoir–associated variables reported by random forest machine learning algorithm analysis. Demographic, clinical, virologic, and immunologic variables are highlighted in gray, green, blue, and orange, respectively, while ART drug families are featured in dark red. Time taking a specific ART family is designated with a “t_” prefix. t_InSTI denotes time taking the integrase strand transfer inhibitors; MeanDecreaseGini is a measure of the importance of a variable based on the Gini Impurity index in the Random Forest algorithm. (**B**) PCA bidimensional plot showing data for LoViReT individuals (<50 HIV-1–DNA copies/10^6^ PBMCs) highlighted in blue and individuals harboring more than 50 HIV-1–DNA copies/10^6^ PBMCs in light gray. The names of the variables representing each arrow are detailed. (**C**) OR estimation and CI based on a logistic regression model for LoViReTs (<50 HIV-1–DNA copies/10^6^ PBMCs). CD4 nadir and CD4 recovery factors are displayed according to a magnitude change of 300 cells/μL, in accordance with the estimated mean of the nadir of the participants from the cohort.

The random forest algorithm ranked each variable according to its importance measure (association) (Supplemental Figure 3), and the 15 variables that were most strongly associated with the LoViReT status were highlighted (Figure 2A). Nadir CD4 (lowest CD4^+^ T cell counts reported throughout the follow-up), zenith pre-ART VL, and CD4 recovery emerged as the 3 factors most closely associated with the LoViReT phenotype. Other relevant factors associated with LoViReTs included the CD4/CD8 ratio at sampling, the time from HIV-1 diagnosis to both the first undetectable VL (timeDtoU) and sampling (timeDtoS), as well as duration of InSTI treatment as part of the ART regimen. Noncategorized analysis was conducted in parallel, yielding similar results (data not shown).

The random forest model identified LoViReT status with an error rate (percentage of misclassified participants) of 30.5% and sensitivity and specificity of 73% and 68.6%, respectively, thus revealing the presence of variables associated with the LoViReT phenotype.

We used cluster-based PCA to characterize the LoViReT group according to the top 15 ranked variables extracted from the random forest model. However, we did not observe a separate, defined cluster for LoViReT individuals in the first components (Figure 2B), suggesting that higher dimensionality or alternative classification methodologies are required.

To further investigate the influence of the variables associated with LoViReT status, we conducted a multiple logistic regression analysis using a forward stepwise selection of all the variables based on the ranking provided by the random forest model.

The variables that were positively associated with being LoViReT were a higher nadir CD4 and CD4/CD8 ratio, lower pre-ART viremia, and shorter time from diagnosis to undetectable VL (timeDtoU) (Figure 2C). Interestingly, we noticed an inverse effect of CD4^+^ T cell recovery from the nadir to sampling counts. This finding suggests that the expansion of the viral reservoir may be due to homeostatic proliferation, which is a surrogate marker of immune reconstitution. Remarkably, in the logistic regression analysis, initiation of ART with InSTIs was mainly associated with LoViReT status, rather than the duration of InSTI treatment. This discrepancy is probably because random forest analysis tends to underestimate the importance of categorical variables. We conducted further analyses to clarify the role of InSTI-containing therapies in the HIV-1 reservoir.

### Initiation of ART with InSTI-containing regimens is associated with lower reservoirs and a shorter time to viral suppression.

**Figure 3:**
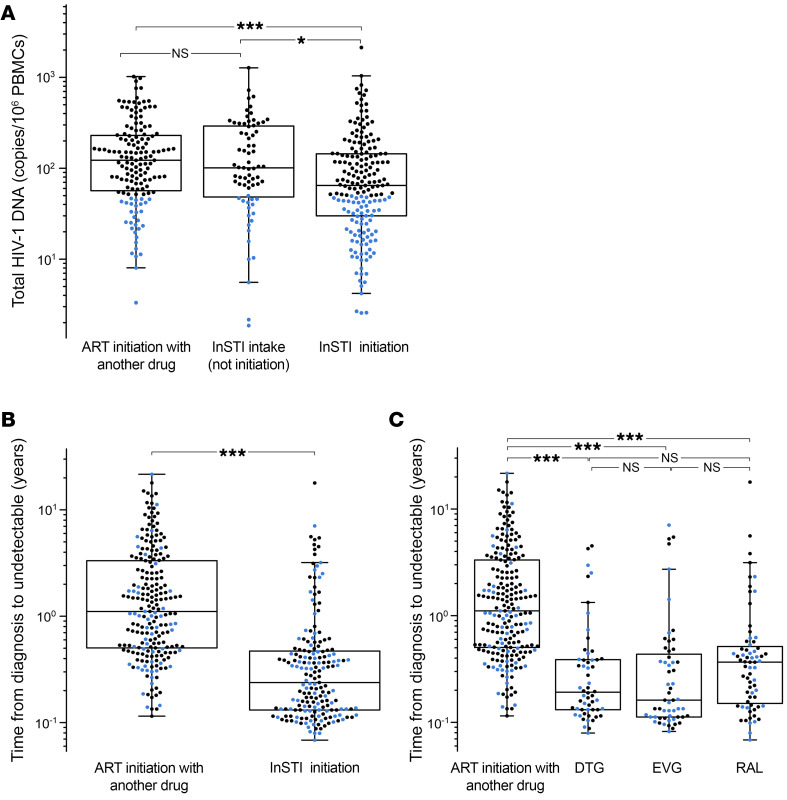
Subanalysis of InSTIs. The effect of initiating ART with InSTI-containing regimens from 2009 on (**A**) the proviral levels quantified in the study population and (**B**) over the time to suppression of the viral load. (**C**) The InSTI-independent effect on the time to suppression was also evaluated considering the various InSTI regimens in the clinical history of our cohort. LoViReT participants (<50 HIV-1–DNA copies/10^6^ PBMCs) are shown in blue. The Mann-Whitney *U* test was used to analyze the effect of InSTI-containing regimens on HIV-1 proviral levels, while Kruskal-Wallis and Conover post hoc tests were used to compare 2 or 3 groups of participants, respectively. NS, *P* ≥ 0.05; *P < 0.05 and ***P < 0.001.

Since the introduction of InSTIs in triple-therapy regimens for ART-naive individuals in 2009, we conducted a subanalysis to examine the effect of InSTI-containing regimens on proviral HIV-1 levels among participants who initiated ART thereafter (from 2009 to 2019). Our analysis revealed that individuals who initiated ART with InSTIs had lower total HIV-1–DNA levels (*P* < 0.001), with LoViReTs accumulating at the bottom of the graph (Figure 3A). In contrast, no such effect was observed among participants who included InSTIs in their regimen following a therapy switch.

We also observed significantly shorter times from diagnosis to undetectable VL after initiation of ART with InSTI-based regimens (*P* < 0.001) (Figure 3B). When differentiating by each integrase inhibitor, we observed that the reduction in this time was independent of whether the initial regimen included dolutegravir, elvitegravir, or raltegravir (Figure 3C).

In summary, our results indicate that the inclusion of InSTIs in initial regimens, combined with later clinical guidelines recommending earlier initiation of treatment (leading to lower immune depletion and higher nadir CD4 counts), affected the HIV-1 reservoir in our PWH population. We conducted additional analyses to further explore the nature of this effect over the past 30 years of ART.

### A decreasing trend in the population’s viral reservoir size following initiation of treatment has been observed since 2007.

To further evaluate the evolution of the established HIV-1 reservoir in the population, we stratified the data by the date of initiation of ART and used a local polynomial regression fitting analysis (locally estimated scatterplot smoothing [LOESS]). We selected the year of ART initiation as a contextual marker to account for the continuous modifications in treatment guidelines that have been gradually implemented over time.

**Figure 4:**
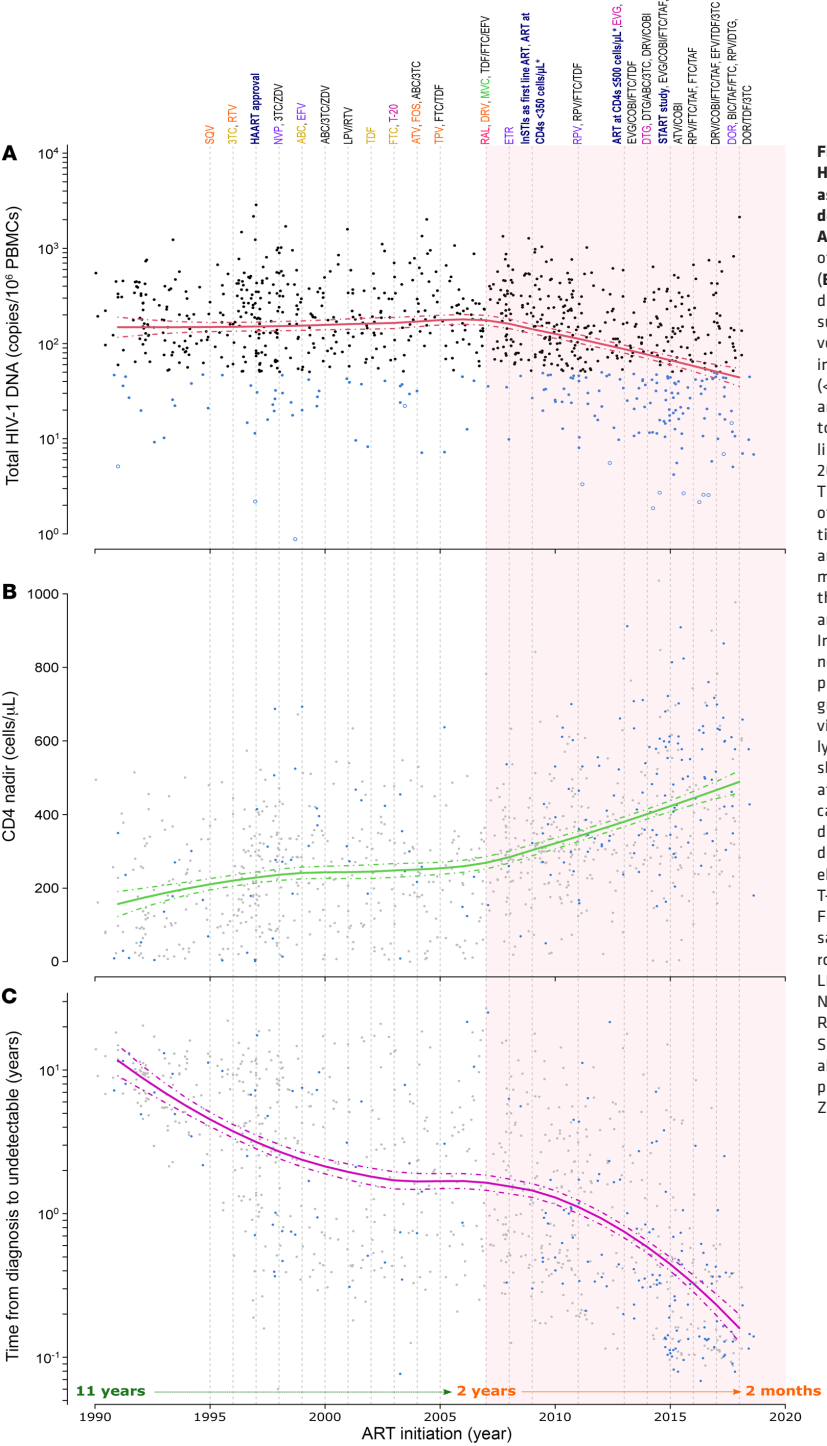
Progression of proviral HIV-1–DNA and low viral reservoir–associated variables over time depending on the participants’ ART initiation date. Distribution of the (**A**) total HIV-1–DNA levels, (**B**) nadir CD4, and (**C**) time from diagnosis to the first viral load suppression data at sampling versus the participants’ ART initiation year. Data on LoViReTs (<50 HIV-1–DNA copies/10^6^ PBMCs) are shown in blue. Open dots refer to values below the detection limit. The biphasic curve area from 2007 is highlighted in pale red. The time course of the approval of several antiretroviral drugs over time is shown above the plot, as are distinct milestones of HIV-1 management (navy blue bold) since the first reported AIDS cases. CCR5 antagonists, fusion inhibitors, InSTIs, nucleoside RT inhibitors, non-nucleoside RT inhibitors, and protease inhibitors are shown in green, magenta, fuchsia, yellow, violet, and orange, respectively, while drug combinations are shown in black. ABC, abacavir; ATV, atazanavir; BIC, bictegravir; CAB, cabotegravir; COBI; cobicistat; DRV, darunavir; DTG, dolutegravir; DOR, doravirine; EFV, efavirenz; EVG, elvitegravir; FTC, emtricitabine; T-20, enfuvirtide; ETR, etravirine; FPV, fosamprenavir; FTR, fostemsavir; HAART, highly active antiretroviral therapy; 3TC, lamivudine; LPV, lopinavir; MVC, maraviroc; NVP, nevirapine; RAL, raltegravir; RPV, rilpivirine; RTV, ritonavir; SQV, saquinavir; TAF, tenofovir alafenamide; TDF, tenofovir disoproxil fumarate; TPV, tipranavir; ZDV, zidovudine.

Our analysis revealed that over the past 30 years, total HIV-1–DNA levels decreased by 0.6 log_10_ (equivalent to a 4-fold decrease) when ART was initiated after 2007 (Figure 4A). This decrease was maintained cumulatively over time, thus explaining the increase in the percentage of LoViReT individuals among the more recently recruited participants. This curve remained similar even when restricting the analysis to participants treated within the IQR of 5–15 years (Supplemental Figure 4).

Various clinical landmarks, such as improvements in HIV-1 treatment guidelines and the introduction of next-generation drugs, are shown in Figure 4. Notably, the introduction of InSTIs occurred simultaneously with the beginning of the decrease in viral reservoir size.

When we examined the plots for other variables associated with the LoViReT phenotype, we observed a nearly 2-fold increase in nadir CD4 counts (Figure 4B) and a 10-fold reduction in the timeDtoU VL (Figure 4C) after 2007, mirroring the trends observed for HIV-1 reservoir size. Notably, the time from diagnosis to viral suppression has undergone a significant change in our population, decreasing from approximately 2.7 years in 1998 to less than 2 months in 2020.

We also observed a significant reduction in the time from diagnosis of HIV-1 to initiation of treatment, with a 24-fold change in the later years (Supplemental Figure 5A). In contrast, for other variables of interest, such as zenith pre-ART VL and the CD4/CD8 ratio, we did not observe similar changes relative to the date of ART initiation (Supplemental Figure 5, B and C). The frequencies of R5/X4 tropism measured in cellular DNA remained relatively stable over time, although the number of missing data points due to difficulties in low reservoir amplification increased in recent years (Supplemental Figure 6).

In summary, we identified a turning point when ART was initiated after 2007, coinciding with changes in various parameters, including the size of the HIV-1 reservoir and some surrogate markers of subsequent treatment guidelines (e.g., nadir CD4 or time from diagnosis of HIV-1 infection to initiation of ART).

## Discussion

Few data have been reported on the effect of evolving guidelines in HIV-1 clinical management on the establishment of the latent HIV-1 reservoir (9–11). Similarly, little information is available on the introduction of next-generation drugs, which are more potent and effective than the original highly active ART approved in 1996 (12).

In this study, we assessed the effect of demographic, clinical, virologic, and immunologic factors on the peripheral blood viral reservoir in a well-characterized cohort of nearly 900 PWH receiving suppressive ART at different time points over the past 3 decades. For this study, we considered the measurement of the HIV-1 reservoir after more than 3 years of suppressed ART as a stable and characteristic value for each individual, as previous studies have reported minimal variations in the latent reservoir even after long-term therapy (13). We found that 20% of individuals (LoViReTs) had extremely low HIV-1–DNA levels. This percentage was more than double the previously observed percentage (9%) in half of the cohort, which comprised only those participants who had received therapy up until 2011 (7). The increase in the proportion of LoViReTs suggests a potential effect of evolving treatment guidelines and more effective antiretroviral drugs on the establishment of the latent HIV-1 reservoir.

To understand the origin of the increased number of participants with low reservoirs over the past decade, we first attempted to identify the main clinical parameters associated with the LoViReT phenotype. Our analysis revealed that higher nadir CD4 counts and CD4/CD8 ratios, together with lower zenith pre-ART VL and shorter timeDtoU VL, were the most significant predictors of LoViReT status. These findings are consistent with those of numerous studies showing that shorter times to initiation of treatment and prevention of CD4^+^ T cell depletion prior to therapy reduce both the reservoir size and its persistence after prolonged ART. This may be due to a limited homeostatic proliferation rate of infected memory CD4^+^ T cells (14) or to preservation of the immune system and circumvention of immune dysregulation, thus limiting constitution of the viral reservoir and favoring its decay (15, 16). Larger CD4 recovery (difference from nadir to sample CD4 levels) was associated with larger reservoirs. This association is supported by the observation that low nadir CD4 counts are related to a large pool of HIV-1–infected cells, primarily owing to an expansion of either latently infected CD4 lymphocytes or a discrete CD4^+^ T cell subpopulation enriched in HIV-1 proviral DNA that replenishes the immune system, even under suppressive ART (17). Thus, it appears that participants with lower nadir CD4 counts who reached higher CD4^+^ T cell counts after initiation of ART were characterized by increased proliferation of latently infected CD4^+^ lymphocytes, resulting in larger HIV-1 reservoirs. Interestingly, this phenomenon may be less apparent when measuring reservoirs in CD4^+^ T cells, as clonal expansion may cause the proportion of infected CD4^+^ T cells to remain constant. The increase in the global reservoir is observed when measured in total PBMCs. Logistic regression analysis provided further evidence that initiation of ART with an InSTI-containing regimen was independently associated with LoViReT status, rather than with the duration of InSTI treatment. Given that a large part of the reservoir in PWH receiving ART is established at initiation of treatment (18), the interruption of the integration step by InSTIs is clearly significant, differentiating this family from other antiretroviral drugs, with wide-ranging implications for the viral reservoir, particularly in terms of its size, even years after initiation of ART (19). The effect of time on InSTIs was only observed in the random forest analysis, which tends to prioritize continuous variables over categorical ones when they are related, as in this instance. Indeed, our in-depth evaluation revealed that individuals initiating ART with InSTI-containing regimens exhibited not only smaller reservoirs, but also shorter times to viral suppression than did those who initiated treatment with another antiretroviral drug class. This effect was independent of the specific InSTI administered, as we observed similar results among participants initiating therapy with raltegravir, dolutegravir, or elvitegravir. It should be noted that, at recruitment, bictegravir was only used in clinical trials and had not yet been initiated as first-line ART in any participant. These findings corroborated the effect of InSTI-containing regimens on the time to viral suppression and suggest that InSTI-based regimens accelerate the process of achieving an undetectable VL (20–25) consistent with recommendations from other groups advocating for prescription of InSTIs as first-line treatment of HIV-1 infection (26) immediately after diagnosis.

We evaluated key reservoir-related parameters using the ART initiation date as a surrogate marker for the complex changes in HIV-1 guidelines over time. Our analysis revealed a biphasic curve in proviral reservoir size, with a notable decline observed from 2007 onward. This trend is mirrored in nadir CD4 counts and the time from diagnosis to undetectable viremia. We believe our study is innovative in that it provides strong evidence that novel clinical guidelines have improved not only the immunological and viral suppression state of PWH, but also had a significant effect on the reservoir size, especially over a specific time period starting in 2007.

The finding of the progressive reduction in HIV-1 reservoirs, first observed in 2007, emphasizes the role of InSTIs in this process. The InSTI raltegravir was approved during the same year, suggesting that this drug family may have contributed to the observed decay in the viral reservoir in our cohort. The virus’s ability to integrate into the genome of PWH initiating InSTI-containing regimens is restricted, thus preventing it from establishing the reservoir and indicating that the dynamics of decay is influenced by the inhibited steps of the virus’s life cycle (27). Further investigation is warranted to elucidate the mechanistic aspects of the apparent effect of InSTIs on reducing HIV-1 reservoirs. Treatment initiation guidelines were therefore modified, shifting from a CD4^+^ T cell count threshold of 250 cells/μL to 350 cells/μL, then 500 cells/μL (28), and, eventually, to the universal treatment policy (29). This is also reflected in the curve, emphasizing the importance of preserving the immune system and limiting the constitution of the viral reservoir by homeostatic proliferation due to CD4^+^ T cell recovery when a low nadir was reached (see above). Although we observed clear variation in these 2 factors, we cannot rule out the possibility of hidden confounders associated with the clinical management and treatment (e.g., single table regimen adherence).

Random forest and logistic regression analyses revealed a correlation between the CD4/CD8 ratio, zenith pre-ART VL, and the LoViReT phenotype. However, these parameters varied minimally over the 30-year period assessed using LOESS regression. This consistency aligns with the understanding that the CD4/CD8 ratio requires an extended period to stabilize (≥1) during treatment, as CD8^+^ T cell counts often remain elevated while CD4^+^ T cell levels gradually normalize (>500 cells/mL). This dynamic leads to a persistently low CD4/CD8 ratio, which is challenging to restore to baseline values (30). With regard to the zenith pre-ART VL, it was expected that there would be little variation in its quantification over time, as this value is typically reported before initiation of treatment or at the viral set point and may not significantly fluctuate based on clinical guidelines. Finally, the PCA was unable to clearly distinguish the LoViReT group, and the factors reported only partly explained the extremely low reservoirs observed in the study population, suggesting that there were other unidentified variables contributing to the proviral quantifications recorded. Further immunovirological analyses are necessary to fully characterize the LoViReT phenotype and identify the complete set of parameters correlated with such proviral quantifications. Additionally, we propose validating these features in additional cohorts to corroborate their importance and compare extreme groups (i.e., individuals with extremely high proviral levels [HiViReTs]) to optimize participant classification in clinical settings.

Our study is subject to a series of limitations. The first significant limitation is the difficulty in collecting and quantifying certain data from the clinical history, such as nadir CD4 counts and zenith pre-ART VLs, given the long follow-up period (since the early 1990s). This may result in overstated values for some individuals, as the entire clinical history is not taken into account. To mitigate this, we analyzed data on a large number of participants with diverse profiles. Second, the lack of additional data of interest, such as viral coinfections, could not be collected for this particular cohort. However, we were able to explore other specific virological factors, including viral tropism, subtype, blips, and viral failures. None of these factors was strongly associated with being LoViReT in a random forest subanalysis with a lower number of individuals (data not shown). The LOESS analysis of HIV-1 tropism revealed a continuous trend over time, with nearly 80% of participants having predominantly CCR5-tropic virus and a persistence of minority CXCR4 tropism within the population. However, after 2005, we observed that the proportion of participants for whom tropism data were missing increased to 20%. This was linked to the higher percentage of PWH with low reservoirs in recent years, which impeded the determination of viral tropism due to the poor efficiency of proviral *env* sequencing in samples with a small reservoir. Third, sex was not predominantly related to lower reservoir size in the multivariate analysis. Age at sampling was a key variable in the random forest analysis but not in the logistic regression analysis, indicating that it was confounded by the time from diagnosis of HIV-1 infection. Fourth, our study used total HIV-1–DNA as a marker of the reservoir, without accounting for the proportion of intact proviruses. Total HIV-1–DNA was used to enable the inclusion of a large number of participants. However, intact proviral DNA assay (IPDA) analysis in a smaller subgroup of participants showed a strong correlation between total HIV-1–DNA and intact proviruses (8), supporting the use of total HIV-1–DNA as a subrogate marker for the bulk reservoir.

In conclusion, this study strongly suggests that recent strategies for the clinical management of HIV-1 infection have significantly affected the establishment of the viral reservoir, particularly since 2007. The combination of new InSTI-based regimens and the preservation of the immune system as a result of earlier, universal treatment at diagnosis were likely the primary drivers of the reductions in reservoir size observed in PWH over the past 2 decades. Our findings highlight the notion that specific clinical parameters may be associated with a reduced HIV-1 reservoir, pointing to optimized clinical management as the first step toward a cure of HIV-1 infection. However, the LoViReT phenotype was not fully determined by clinical factors alone, indicating that these individuals are exceptional models for unraveling novel immunovirological mechanisms associated with low viral reservoirs. These mechanisms could potentially be mimicked in larger populations of PWH to further advance toward a cure.

## Methods

### Sex as a biological variable.

Our study included both men and women (described in Table 1). The sex of the participants was self-reported.

### Study participants.

We analyzed 892 participants undergoing regular follow-up at the Hospital Germans Trias i Pujol (*n* = 760) and the Hospital Clinic (*n* = 132) in Barcelona. Data from 451 of these participants have been reported elsewhere (7). All the individuals fulfilled the inclusion criterion, namely, they had been receiving ART with undetectable viremia (<50 copies HIV-1-RNA/μL plasma) for at least 3 years. Demographic and clinical data were collected from the clinical database.

### Quantification of total HIV-1–DNA.

We measured the HIV-1 reservoir in participants who had been on suppressive ART for more than 3 years and considered the resulting value a constant for each participant. This criterion was based on the notion that the reservoir decays and eventually stabilizes after the first few years of ART, showing minimal variation despite long-term ART, as recently demonstrated (13, 31). Viral reservoir size was evaluated using lysed PBMC extracts to quantify total HIV-1–DNA by droplet digital PCR (ddPCR) (Bio-Rad), as previously reported (32). Proviral quantification for each individual corresponded to the highest value obtained from the 2 primer sets used (5′ long terminal repeats [LTRs] or *gag*). LoViReT individuals were defined as having fewer than 50 total HIV-1–DNA copies/10^6^ PBMCs.

### Viral tropism and subtyping.

Viral coreceptor tropism and subtype were assessed on proviral DNA by sequencing HIV-1 *gp120* hypervariable region 3 (V3) and subsequently predicted through the Geno2Pheno algorithm (GENAFOR) (33). Tropism was inferred using the Genotypic Prediction of Coreceptor Usage tool with a false-positive rate (FPR) of 10% (non-R5 tropism: FPR ≤10%), whereas the HIV-1 subtype was estimated using the Geno2Pheno Virus Detection and Subtyping Tool.

### Clinical variables.

We studied 20 raw and derived variables (Table 1) and categorized 30 different antiretroviral drugs into 6 families (Supplemental Table 1).

Since information was sometimes lacking during the pre-ART period, we estimated the nadir CD4 as the minimum CD4^+^ T cell count/μL reported in the clinical history. Zenith pre-ART VL was defined as the maximum pre-ART VL (in log_10_ copies/mL plasma). Participants with missing pre-ART data were excluded from any analysis involving this specific variable (*n* = 67). The remaining variables examined (e.g., AUC VL, viral blips, and failures) were also analyzed but not reported in the results, as they were not associated with the LoViReT phenotype in the multivariate analysis (data not shown).

Regarding treatment regimens, we also evaluated the time on treatment with each ART drug (*n* = 30) reported from the clinical history and recorded an indicator of whether the individual had started treatment with each drug or not (dichotomous variable). These analyses were also conducted by grouping the drugs into families (*n* = 6) (Supplemental Table 1).

### Statistics.

Random forest analysis was performed to detect features associated with LoViReT status based on a list of 20 raw variables and variables derived from clinical histories and categorized drugs into 30 different antiretroviral drugs from 6 families. Random forest analysis was chosen, since it is a powerful, versatile ensemble learning method that integrates independently adjusted multiple models. Random forest analysis is based on a set of decision trees, each trained on a different subset of the training data, which are combined (bootstrap aggregation) while introducing randomness into their fit in order to add diversity to the set. Although this model prioritizes a continuous variable over a categorical one if the two are correlated, it presents multiple advantages, making it an attractive choice for our study. The random forest model provided a ranking of variables according to their relative importance, which allowed us to identify a set of 15 relevant variables based on the distribution of the importance measure. The top 15 phenotype-associated LoViReT variables were chosen, albeit subjectively, on the basis of changes in the continuity of the distribution of the importance measure (similar to the Elbon selection process). Random forest tuning parameters were adjusted to offset unbalanced group sizes.

To explore and describe correlations and associations between variables, correlograms and principal component analysis (PCA) were used. Correlations and the estimated effect of selected features were also analyzed by fitting a multivariate logistic regression model on the basis of the importance ranking provided by the random forest analysis and following a forward stepwise selection of variables using the likelihood ratio test.

In addition, a subanalysis of the effect of InSTI-containing regimens on HIV-1 proviral levels was performed using the Mann-Whitney *U* test. The Kruskal-Wallis test and Conover post hoc test were used to compare 2 and 3 groups of participants, respectively.

Our results highlight changes in clinical variables depending on the year in which ART was initiated. We analyzed and graphed the variables using LOESS. The analyses were performed with R (version 4.3.1), with a *P* value of less than 0.05 considered statistically significant.

### Study approval.

All participants provided their signed informed consent. The study was approved by the ethics committee at both recruiting centers (Hospital Germans Trias i Pujol and Hospital Clinic in Barcelona; reference: PI-14-083).

### Data availability.

Study data are not publicly available to preserve the privacy of the research participants. Data can be made available by the corresponding authors to any interested researcher following approval of a concept sheet summarizing the analysis to be performed. Deidentified participant data and a data dictionary can be made available and shared under a data transfer agreement. Requests for access to the LoViReT study data should be sent to the corresponding authors.

## Author contributions

According to the CRediT taxonomy, IGN, JMP, and MS contributed to the study’s conceptualization, data curation, formal analysis, funding acquisition, investigation, methodology, project administration, resources, software, supervision, validation, visualization and to the writing of the original draft and review and editing of the final version of the manuscript. All authors accessed and verified the data (with VU, CG, BM) and were responsible for the decision to submit the manuscript. VU contributed to data curation, formal analysis, methodology, software, validation, visualization, and writing of the original draft and review and editing of the manuscript. CG, MDCGG, SML, MCP, and EG contributed to investigation, resources, and writing, reviewing, and editing of the manuscript. BM, LB, CM, FG, and LL contributed to the resources, supervision, and writing, reviewing and editing of the manuscript. BC contributed to the resources and writing, reviewing, and editing of the manuscript. LV contributed to the supervision and writing, reviewing, and editing of the manuscript. VCM contributed to the supervision and writing, reviewing, and editing of the manuscript. RPS contributed to funding acquisition, supervision, and writing, reviewing, and editing of the manuscript. The order of the names of the co–first authors and co–last authors was decided by internal agreements between the authors.

## Supplementary Material

Supplemental data

ICMJE disclosure forms

Supporting data values

## Figures and Tables

**Table 1:**
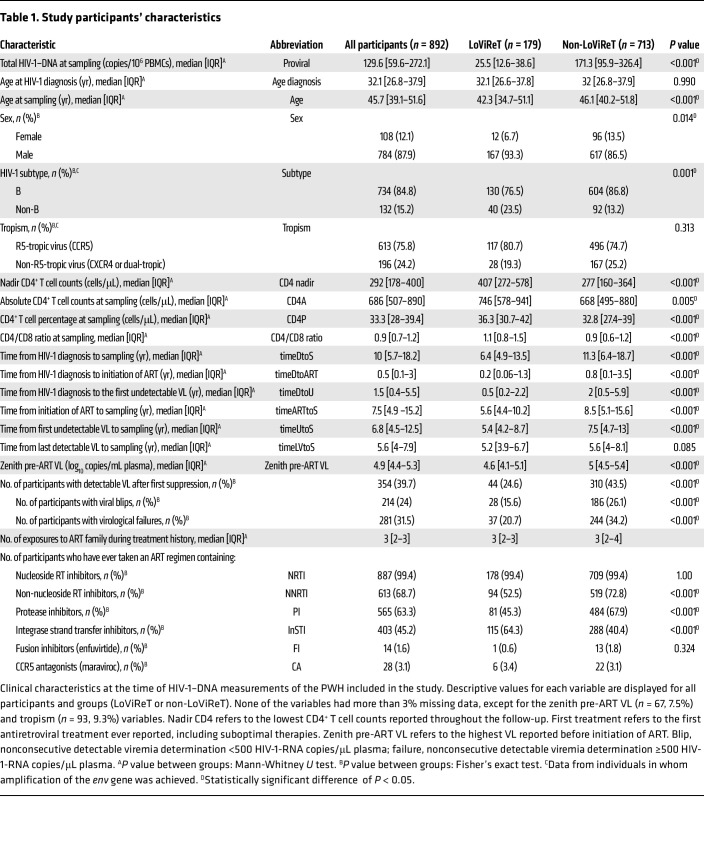
Study participants’ characteristics

## Version 1 11/29/2024

In-Press Preview

## Version 2 01/16/2025

Electronic publication
