# Supplementary material for: Assessing advances in three decades of clinical antiretroviral therapy on the HIV-1 reservoir
Source: J Clin Invest. 2024 Nov 29;135(2):e183952. doi: 10.1172/JCI183952 (PMC11735095; doi:10.1172/JCI183952)
Supplement: Supplemental data [file jci-135-183952-s251.pdf]

Supplementary Materials

González-Navarro et al.

Supplemental Table 1. ART drugs. List of the 30 antiretroviral drugs evaluated to determine their association with proviral quantifications in the study cohort. ART drug approval dates were extracted from the *Centro de información online de medicamentos de la* AEMPS (CIMA) online open sources available at https://cima.aemps.es/cima/publico/home.html (accessed 30.05.2024). Abbreviations: *Agencia Española de Medicamentos y Productos Sanitarios*, AEMPS (Spanish Agency for Medicines and Medical Devices).

**Family**	**Generic name**	**Abbreviation**	**AEMPS approval date** (dd/mm/yyyy)
Nucleoside reverse transcriptase inhibitors	Abacavir	ABC	08/07/1999
	Emtricitabine	FTC	02/12/2003
	Lamivudine	3TC	01/11/1996
	Tenofovir disoproxil fumarate	TDF	25/02/2002
	Tenofovir alafenamide	TAF	25/01/2017
	Zidovudine (Azidothymidine)	ZDV (AZT)	01/07/1988
	Stavudine	D4T	08/05/1996
	Didanosine	DDI	11/08/2000
Non-nucleoside reverse transcriptase inhibitors	Doravirine	DOR	08/02/2019
	Efavirenz	EFV	28/05/1999
	Etravirine	ETR	16/12/2011
	Nevirapine	NVP	05/02/1998
	Rilpivirine	RPV	13/01/2012
	Zalcitabine	DDC	29/03/1994
Protease inhibitors	Atazanavir	ATV	29/03/2004
	Darunavir	DRV	16/02/2009
	Fosamprenavir	FPV	22/07/2004
	Ritonavir	RTV	26/08/1996
	Tipranavir	TPV	25/10/2005
	Nelfinavir	NFV	22/01/1998
	Saquinavir	SQV	04/10/1996
	Indinavir	IND	04/10/1996
	Lopinavir	LPV	13/06/2001
	Amprenavir	APV	20/10/2000
Integrase strand transfer inhibitors	Dolutegravir	DTG	12/02/2014
	Raltegravir	RAL	21/12/2007
	Bictegravir	BIC	10/07/2018
	Elvitegravir	EVG	26/06/2013
Fusion inhibitors	Enfuvirtide	T-20	05/06/2003
CCR5 antagonists	Maraviroc	MVC	27/09/2007

Supplemental Figure 1. Linear regression. Correlation for the variables studied in the linear regression analysis. Colors represent the correlation between the variables; positively related factors are shown in blue, while negatively related factors are shown in red. Dot size indicates the degree of correlation; the larger the size, the stronger the correlation between the 2 variables. Abbreviations: ART, antiretroviral therapy; InSTI, integrase strand transfer inhibitor; NRTI, nucleoside reverse transcriptase inhibitor; NNRTI, non-nucleoside reverse transcriptase inhibitor; PI, protease inhibitor; VL, viral load.

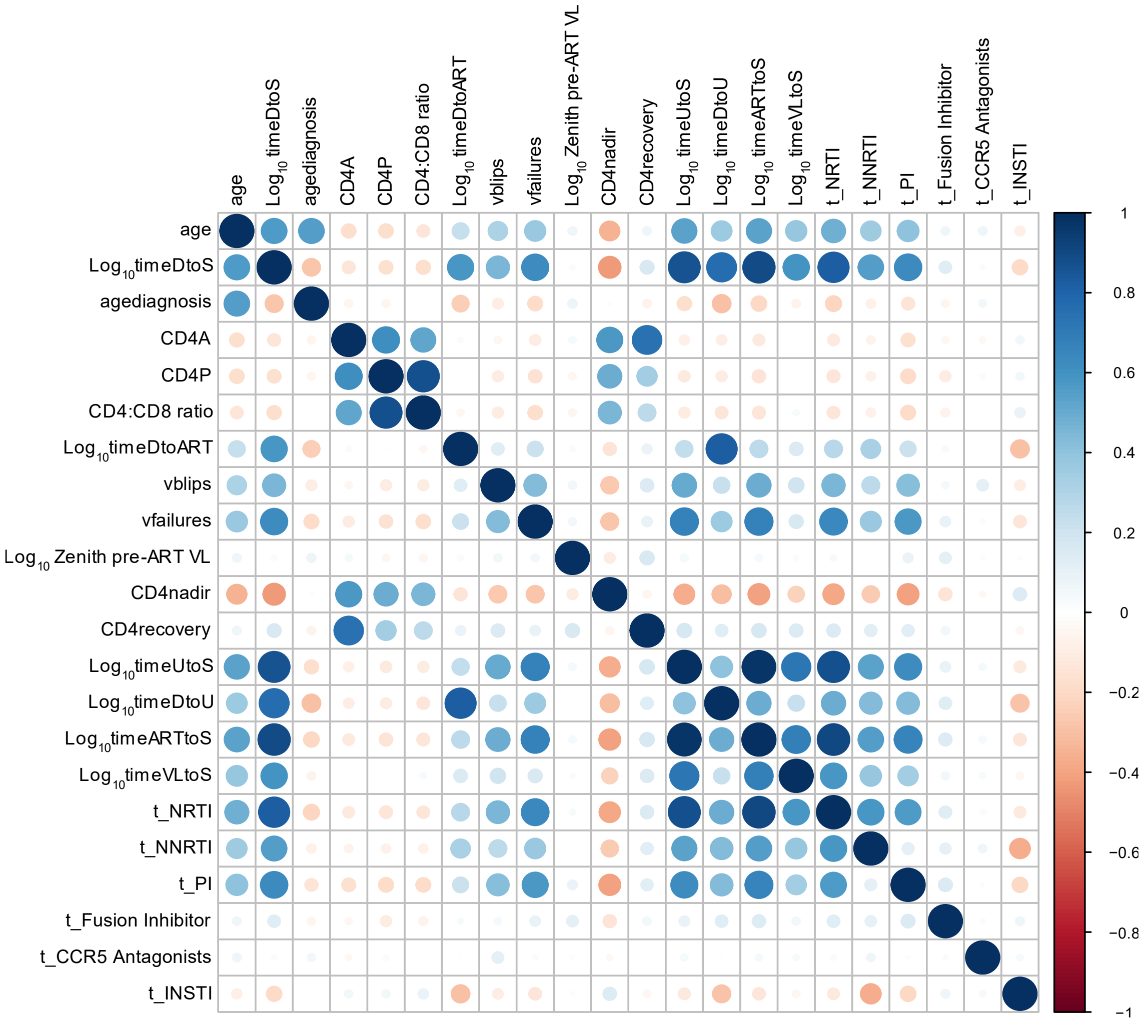

Supplemental Figure 2. Time-related factors associated with LoViReT status. Graphic description of the periods that correspond to each time-related factor evaluated. Viral load blips refer to non-consecutive detectable viremia <500 HIV-1-RNA copies/µL of plasma, while failures are defined as a single, specific positive viremia determination with a maximum peak ≥500 HIV-1-RNA copies/µL of plasma. Abbreviations: ART, antiretroviral therapy; D, diagnosis; LV, last viremia; S, sampling; U, undetectable.

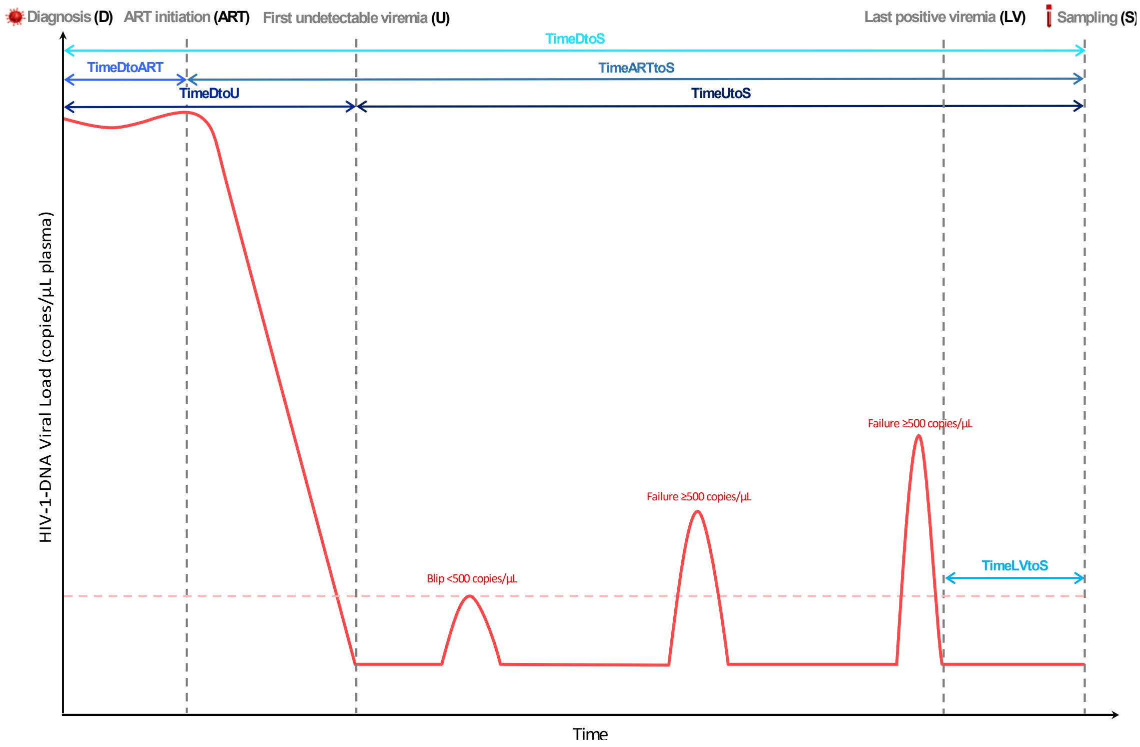

Supplemental Figure 3. Random forest analysis. Importance measure–based classification of the complete set of variables (n = 20) and treatment regimens (30 drugs from 6 distinct families) evaluated using the random forest approach. Demographic, clinical, virologic, and immunologic variables are highlighted in gray, green, blue, and orange, respectively, while drug regimens are featured in violet (ART drugs as backbone therapy) and dark red (ART families). Initiation of and time on a specific ART drug (light violet) or family-regimen (light red) are prefixed by "t_" and "i_", respectively. Abbreviations: ABC, abacavir; APV, amprenavir; ATV, atazanavir; BIC, bictegravir; DRV, darunavir; DDI, didanosine; DDC, dideoxycytidine; DTG, dolutegravir; DOR, doravirine; EFV, efavirenz; EVG, elvitegravir; FTC, emtricitabine; T-20, enfuvirtide; ETR, etravirine; FPV, fosamprenavir; IND, indinavir; InSTI, integrase strand transfer inhibitor; 3TC, lamivudine; LPV, lopinavir; MVC, maraviroc; NFV, nelfinavir; NRTI, nucleoside reverse transcriptase inhibitor; NNRTI, non-nucleoside reverse transcriptase inhibitor; NVP, nevirapine; RAL, raltegravir; RPV, rilpivirine; RTV, ritonavir; SQV, saquinavir; D4T, stavudine; TAF, tenofovir alafenamide; TDF, tenofovir disoproxil fumarate; TPV, tipranavir; VL, viral load; ZDV, zidovudine.

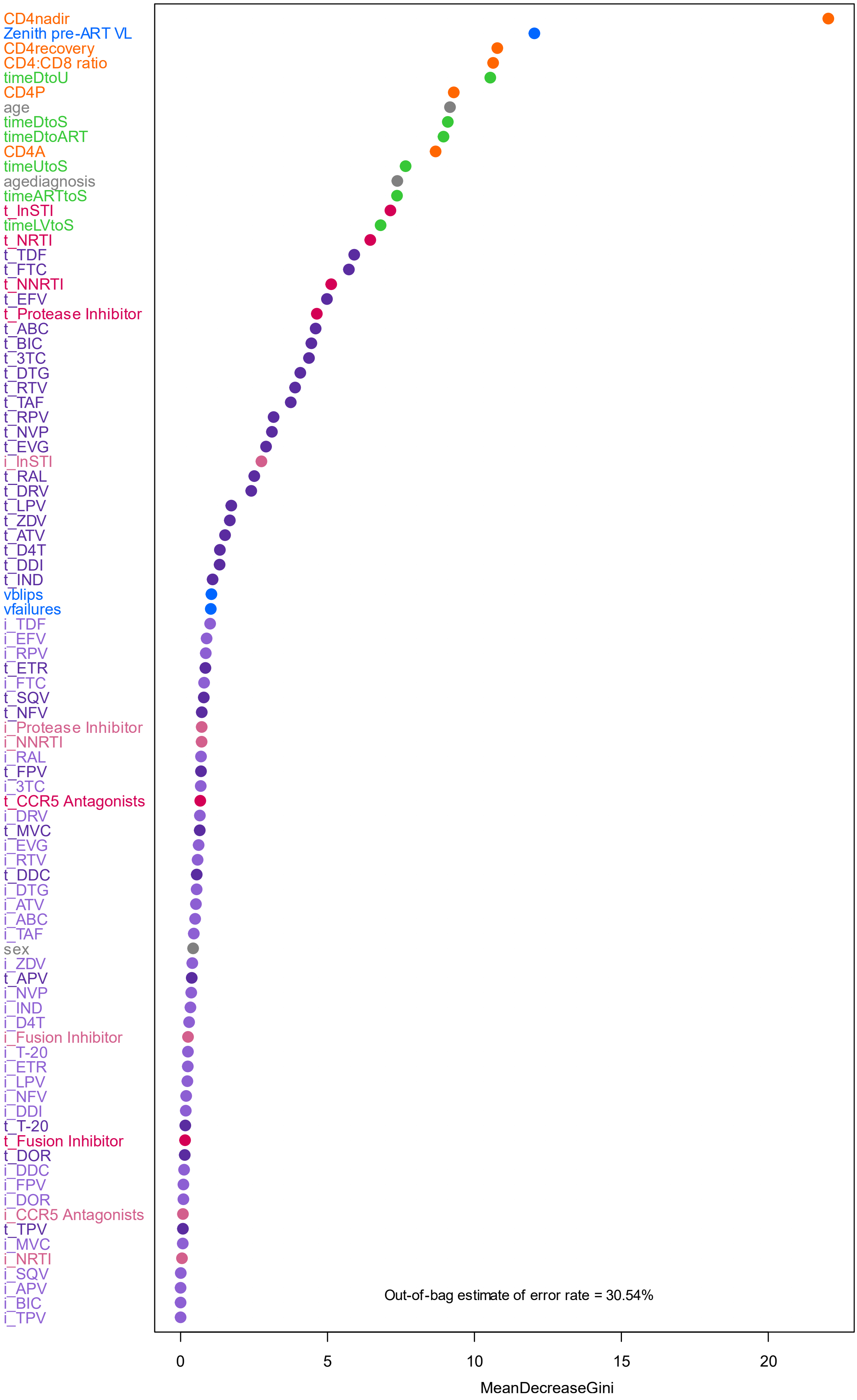

CD4nadir
Zenith pre-ART VL
CD4recovery
CD4:CD8 ratio
timeDtoU
CD4P
age
timeDtoS
timeDtoART
CD4A
timeUtoS
agediagnosis
timeARTtoS
t_InSTI
timeLVtoS
t_NRTI
t_TDF
t_FTC
t_NNRTI
t_EFV
t_Protease Inhibitor
t_ABC
t_BIC
t_3TC
t_DTG
t_RTV
t_TAF
t_RPV
t_NVP
t_EVG
i_InSTI
t_RAL
t_DRV
t_LPV
t_ZDV
t_ATV
t_D4T
t_DDI
t_IND
vblips
vfailures
i_TDF
i_EFV
i_RPV
t_ETR
i_FTC
t_SQV
t_NFV
i_Protease Inhibitor
i_NNRTI
i_RAL
t_FPV
i_3TC
t_CCR5 Antagonists
i_DRV
t_MVC
i_EVG
i_RTV
t_DDC
i_DTG
i_ATV
i_ABC
i_TAF
sex
i_ZDV
t_APV
i_NVP
i_IND
i_D4T
i_Fusion Inhibitor
i_T-20
i_ETR
i_LPV
i_NFV
i_DDI
t_T-20
t_Fusion Inhibitor
t_DOR
i_DDC
i_FPV
i_DOR
i_CCR5 Antagonists
t_TPV
i_MVC
i_NRTI
i_SQV
i_APV
i_BIC
i_TPV
Out-of-bag estimate of error rate = 30.54%
0
5
10
15
20
MeanDecreaseGini

Supplemental Figure 4. Progression of proviral HIV-1-DNA over time depending on participants' ART initiation date, narrowed down to the participants of 5-15 years under ART. Distribution of the total HIV-1-DNA levels at sampling versus participants' ART initiation year only for the participants within the interquartile range of time under ART, meaning the ones treated from 5 to 15 years (n=425). LoViReTs (<50 HIV-1-DNA copies/10^6 PBMCs) are shown in blue. Open dots refer to values below the detection limit. The bi-phasic curve area from 2007 is highlighted in light red, being very similar to when we used all the participants. The time course of the approval of several antiretroviral drugs over time is shown above the plot, as are distinct milestones on HIV-1 management (navy blue bold) since the first reported AIDS cases. CCR5 antagonists, fusion inhibitors, InSTIs, nucleoside reverse transcriptase inhibitors, non-nucleoside reverse transcriptase inhibitors, and protease inhibitors are shown in green, magenta, fuchsia, yellow, violet, and orange, respectively, while drug combinations are shown in black. Abbreviations: ABC, abacavir; ATV, atazanavir; BIC, bictegravir; CAB, cabotegravir; COBI; cobicistat; DRV, darunavir; DTG, dolutegravir; DOR, doravirine; EFV, efavirenz; EVG, elvitegravir; FTC, emtricitabine; T-20, enfuvirtide; ETR, etravirine; FPV, fosamprenavir; FTR, fostemsavir; HAART, highly active antiretroviral therapy; 3TC, lamivudine; LPV, lopinavir; MVC, maraviroc; NVP, nevirapine; RAL, raltegravir; RPV, rilpivirine; RTV, ritonavir; SQV, saquinavir; TAF, tenofovir alafenamide; TDF, tenofovir disoproxil fumarate; TPV, tipranavir; ZDV, zidovudine.

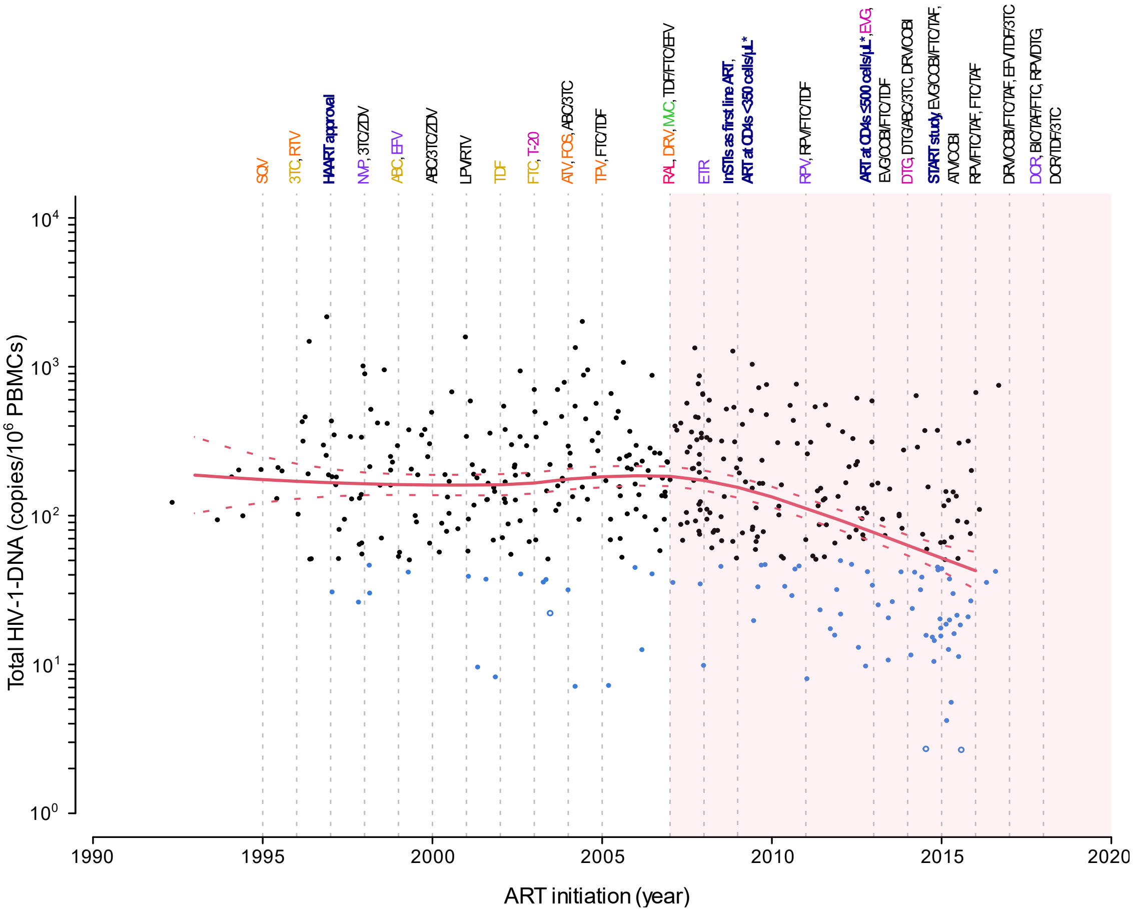
SQV
3TC, RTV
HAART approval
NVP, 3TC/ZDV
ABC, EFV
ABC/3TC/ZDV
LPV/RTV
TDF
FTC, T-20
ATV, FOS, ABC/3TC
TPV, FTC/TDF
RAL, DRV, MVC, TDF/FTC/EFV
ETR
InSTIs as first line ART,
ART at CD4s <350 cells/μL*
RPV, RPV/FTC/TDF
ART at CD4s ≤500 cells/μL*, EVG,
EVG/COBI/FTC/TDF
DTG, DTG/ABC/3TC, DRV/COBI
START study, EVG/COBI/FTC/TAF,
ATV/COBI
RPV/FTC/TAF, FTC/TAF
DRV/COBI/FTC/TAF, EFV/TDF/3TC
DOR, BIC/TAF/FTC, RPV/DTG,
DOR/TDF/3TC
Total HIV-1-DNA (copies/10^6 PBMCs)
10^0
10^1
10^2
10^3
10^4
1990
1995
2000
2005
2010
2015
2020
ART initiation (year)

Supplemental Figure 5. Progression of additional low viral reservoir-associated variables depending on participants' ART initiation date. Distribution of the (**A**) time from diagnosis to initiation of treatment, (**B**) Zenith pre-ART viral load, and (**C**) CD4:CD8 ratio data at sampling versus participants' year of initiation of ART. LoViReTs (<50 HIV-1-DNA copies/10^6 PBMCs) are shown in blue. Open dots refer to values below the detection limit. The bi-phasic curve area from 2007 is shown in light red. The time course for development of several antiretroviral drugs and distinct milestones in HIV-1 management (navy blue bold) since the first reported AIDS cases are shown at the top of the figure. CCR5 antagonists, fusion inhibitors, InSTIs, nucleoside reverse transcriptase inhibitors, non-nucleoside reverse transcriptase inhibitors, and protease inhibitors are shown in green, magenta, fuchsia, yellow, violet, and orange, respectively, while drug combinations are shown in black. Abbreviations: ABC, abacavir; ATV, atazanavir; BIC, bictegravir; CAB, cabotegravir; COBI; cobicistat; DRV, darunavir; DTG, dolutegravir; DOR, doravirine; EFV, efavirenz; EVG, elvitegravir; FTC, emtricitabine; T-20, enfuvirtide; ETR, etravirine; FPV, fosamprenavir; FTR, fostemsavir; HAART, highly active antiretroviral therapy; 3TC, lamivudine; LPV, lopinavir; MVC, maraviroc; NVP, nevirapine; RAL, raltegravir; RPV, rilpivirine; RTV, ritonavir; SQV, saquinavir; TAF, tenofovir alafenamide; TDF, tenofovir disoproxil fumarate; TPV, tipranavir; ZDV, zidovudine.

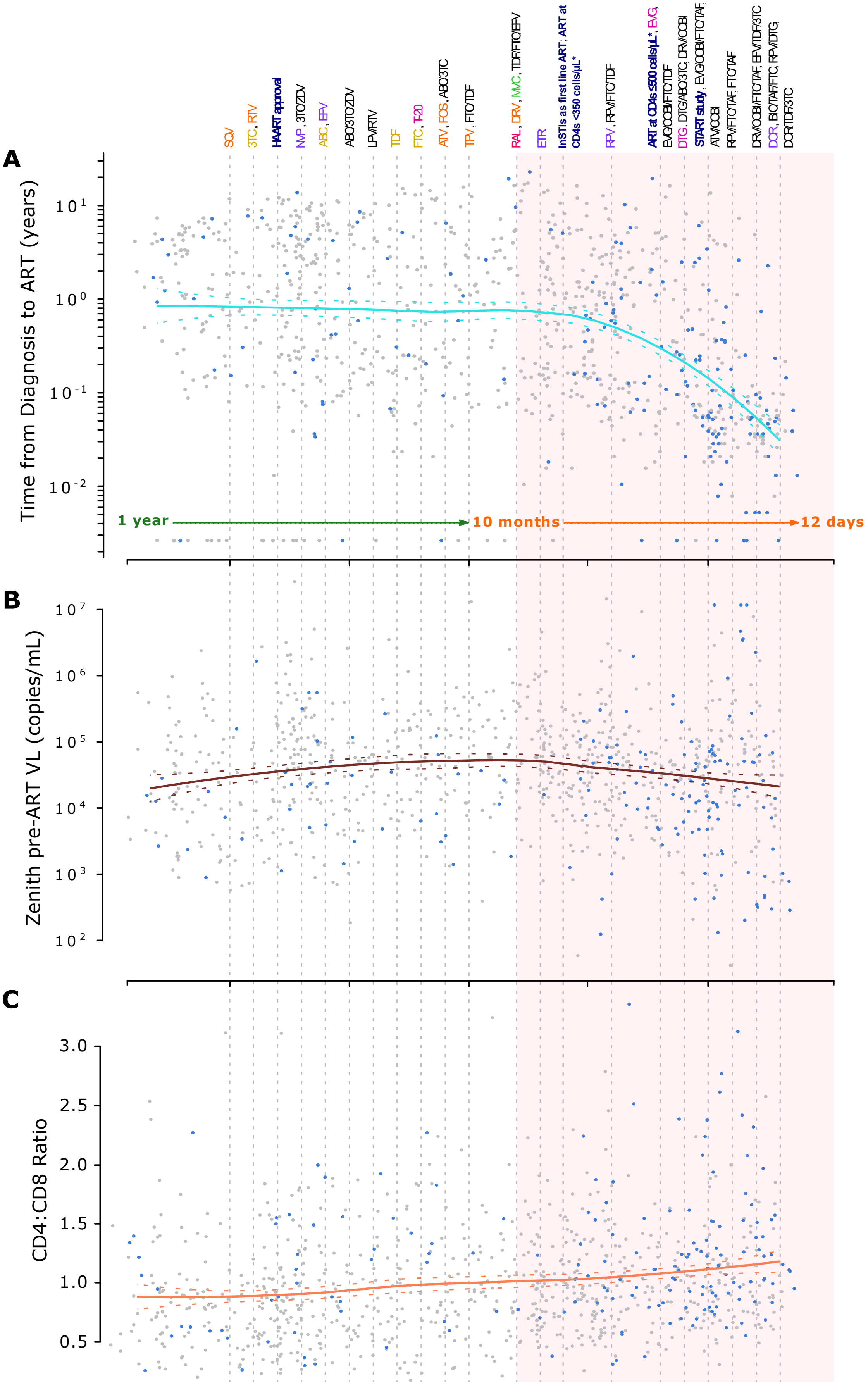

SQV
3TC, RTV
HAART approval
NVP, 3TC/ZDV
ABC, EFV
ABC/3TC/ZDV
LPV/RTV
TDF
FTC, T-20
ATV, FOS, ABC/3TC
TPV, FTC/TDF
RAL, DRV, MVC, TDF/FTC/EFV
ETR
InSTIs as first line ART; ART at CD4s <350 cells/μL*
RPV, RPV/FTC/TDF
ART at CD4s ≤500 cells/μL*, EVG, EVG/COBI/FTC/TDF
DTG. DTG/ABC/3TC, DRV/COBI
START study, EVG/COBI/FTC/TAF, ATV/COBI
RPV/FTC/TAF, FTC/TAF
DRV/COBI/FTC/TAF, EFV/TDF/3TC
DOR, BIC/TAF/FTC, RPV/DTG, DOR/TDF/3TC
A
Time from Diagnosis to ART (years)
10^{1}
10^{0}
10^{-1}
10^{-2}
1 year
10 months
12 days
B
Zenith pre-ART VL (copies/mL)
10^{7}
10^{6}
10^{5}
10^{4}
10^{3}
10^{2}
C
CD4:CD8 Ratio
3.0
2.5
2.0
1.5
1.0
0.5
1990
1995
2000
2005
2010
2015
2020
ART initiation (year)

Supplemental Figure 6. Changes in tropism over time. Trend in participants' viral tropism according to the percentage of PWH who are either CCR5-tropic (green) or CXCR4-tropic (red) per year. The proportion of missing data is represented in gray.

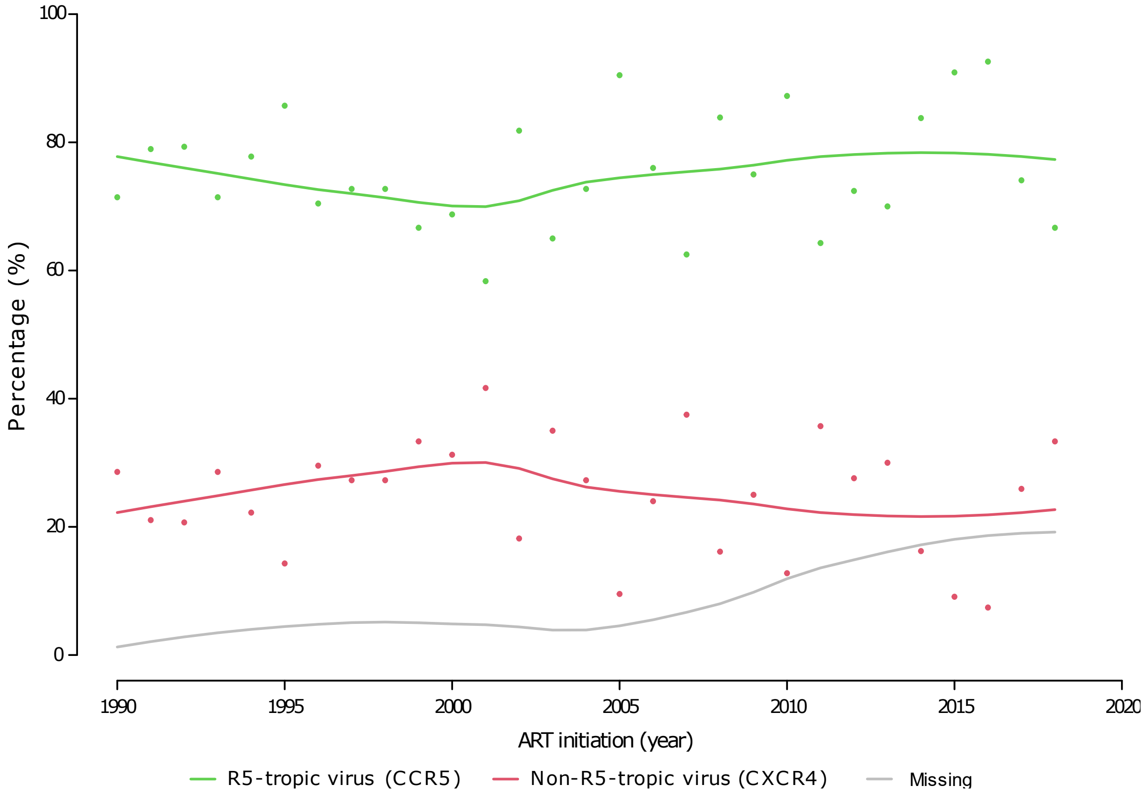

Supplemental Acknowledgements

Reversing Immune Dysfunction for HIV-1 Eradication (RID-HIV) Martin Delaney Collaboratory group

In addition to the authors of this article, members of the Reversing Immune Dysfunction for HIV-1 Eradication (RID-HIV) Collaboratory group include:

- Sumit Chanda[1]

  [1]Department of Immunology and Microbiology, The Scripps Research Institute, La Jolla, CA, USA

- Paula Cannon[2]

  [2]Department of Molecular Microbiology and Immunology, Keck School of Medicine of the University of Southern California , Los Angeles, CA, USA

- Jeff Taylor[3,4]

  [3]UCSD AntiViral Research Center Community Advisory Board, San Diego, CA, USA

  [4]HIV+Aging Research Project-Palm Springs (HARP-PS), Palm Springs, CA, USA

- Nicholas Cosford[5]

  [5]Sanford Burnham Prebys Medical Discovery Institute, La Jolla, CA, USA

- Thomas Hope[6]

  [6]Cell and Developmental Biology, Feinberg School of Medicine, Northwestern University, Chicago, IL, USA

- Bonnie Howell[7]

  [7]MRL, Merck & Co., Inc., Rahway, NJ, USA

- Daphne Ma[7]

  [7]MRL, Merck & Co., Inc., Rahway, NJ, USA

- Jeffrey S. Miller[8]

  [8]Department of Medicine, University of Minnesota, Minneapolis, MN, USA

- Lars Pache[9]

  [9]NCI Designated Cancer Center, Sanford Burnham Prebys Medical Discovery Institute, La Jolla, CA, USA

- Mirko Paiardini[10,11]

  [10]Division of Microbiology and Immunology, Emory National Primate Research Center, Emory University, Atlanta, GA, USA

  [11]Department of Pathology and Laboratory Medicine, Emory University School of Medicine, Atlanta, GA, USA

- Alan S. Perelson[12,13]

  [12]Theoretical Biology and Biophysics, Los Alamos National Laboratory, Los Alamos, NM, USA

  [13]Santa Fe Institute, Santa Fe, NM, USA

- Constantinos Petrovas[14,15]

  [14]Tissue Analysis Core, Immunology Laboratory, Vaccine Research Center, NIAID, NIH, Bethesda, MD, USA

  [15]Department of Laboratory Medicine and Pathology, Institute of Pathology, Lausanne University Hospital and Lausanne University, Lausanne, Switzerland

- Vicente Planelles[16]

[16]Department of Pathology, Spencer Fox Eccles School of Medicine, University of Utah, Salt Lake City, UT, USA

- Ruy M. Ribeiro[17]

[17]Theoretical Biology and Biophysics, Los Alamos National Laboratory, Los Alamos, NM, USA

- Susan P. Ribeiro[18,19,20]

[18]Emory Vaccine Center, Emory University School of Medicine, Atlanta, GA, USA

[19]Pathology Advanced Translational Research Unit (PATRU), Department of Pathology and Laboratory Medicine, Emory University School of Medicine, Atlanta, GA, USA

[20]Winship Cancer Institute, Emory University School of Medicine, Atlanta, GA, USA

- Ashish A. Sharma[19]

[19]Pathology Advanced Translational Research Unit (PATRU), Department of Pathology and Laboratory Medicine, Emory University School of Medicine, Atlanta, GA, USA

- Kalpit Vora[20]

[20]Infectious Diseases and Vaccines Research, Merck & Co., Inc., West Point, PA, USA

- Ross Wilson[21,22,23]

[21]Innovative Genomics Institute, University of California Berkeley, Berkeley, CA, USA

[22]Department of Molecular and Cell Biology, University of California Berkeley, Berkeley, CA, USA

[23]California Institute for Quantitative Biosciences at University of California Berkeley, Berkeley, CA, USA
